# Ticks and associated pathogens in camels (*Camelus dromedarius*) from Riyadh Province, Saudi Arabia

**DOI:** 10.1186/s13071-020-3973-y

**Published:** 2020-02-28

**Authors:** Abdullah D. Alanazi, Viet Linh Nguyen, Mohamed S. Alyousif, Ranju R. S. Manoj, Abdulaziz S. Alouffi, Ridolfi Donato, Alireza Sazmand, Jairo A. Mendoza-Roldan, Filipe Dantas-Torres, Domenico Otranto

**Affiliations:** 1 Department of Biological Sciences, Faculty of Science and Humanities, Shaqra University, Ad-Dawadimi, Saudi Arabia; 2 Dipartimento di Medicina Veterinaria, Università degli Studi di Bari, Bari, Italy; 3 Department of Zoology, College of Science, King Saud University, Riyadh, Saudi Arabia; 4 Life Science and Environment Sector, King Abdulaziz City for Science and Technology, Riyadh, Saudi Arabia; 5 Istituto Zooprofilattico della Puglia e della Basilicata, Bari, Italy; 6 Department of Pathobiology, Faculty of Veterinary Science, Bu-Ali Sina University, Hamedan, Iran; 7 Department of Immunology, Oswaldo Cruz Foundation, Aggeu Magalhães Institute, Recife, Pernambuco Brazil

**Keywords:** Ticks, Tick-borne pathogens, Camels, Saudi Arabia, *Anaplasma platys*, *Anaplasma phagocytophilum*, *Ehrlichia canis*, *Hepatozoon canis*

## Abstract

**Background:**

Camel production in Saudi Arabia is severely affected by various diseases and by inadequate veterinary services. Ticks and tick-borne pathogens (TBPs) affect the health and wellbeing of camels consequently diminishing their productivity and performances. In addition, camels may act as hosts for TBPs (e.g. *Anaplasma phagocytophilum*) causing diseases in humans. The current study aimed to determine the prevalence of ixodid ticks and molecularly investigate the associated pathogens in camels from Saudi Arabia.

**Methods:**

Blood and tick samples were collected from camels (*n* = 170) in Riyad Province of Saudi Arabia. Ticks were morphologically identified, and blood of camels were molecularly screened for apicomplexan (i.e. *Babesia* spp., *Theileria* spp., *Hepatozoon* spp.) and rickettsial parasites (i.e. *Ehrlichia* spp. and *Anaplasma* spp.).

**Results:**

Of the 170 camels examined, 116 (68.2%; 95% CI: 60.9–75.1%) were infested by ticks with a mean intensity of 2.53 (95% CI: 2.4–2.6). In total of 296 ticks collected, *Hyalomma dromedarii* was the most prevalent (76.4%), followed by *Hyalomma impeltatum* (23.3%) and *Hyalomma excavatum* (0.3%). Of the tested animals, 13 (7.6%; 95% CI: 4.3–12.8%) scored positive to at least one TBP, with *Anaplasma platys* (5.3%; 95% CI: 2.7–9.9%) being the most prevalent species, followed by *Anaplasma phagocytophilum*, *Anaplasma* sp., *Ehrlichia canis* and *Hepatozoon canis* (0.6% each; 95% CI: 0.04–3.4%). None of the camels were found to be co-infected with more than one pathogen. All samples tested negative for *Babesia* spp. and *Theileria* spp.

**Conclusions:**

The present study reveals the occurrence of different tick species and TBPs in camels from Saudi Arabia. Importantly, these camels may carry *A. phagocytophilum* and *A. platys*, representing a potential risk to humans.
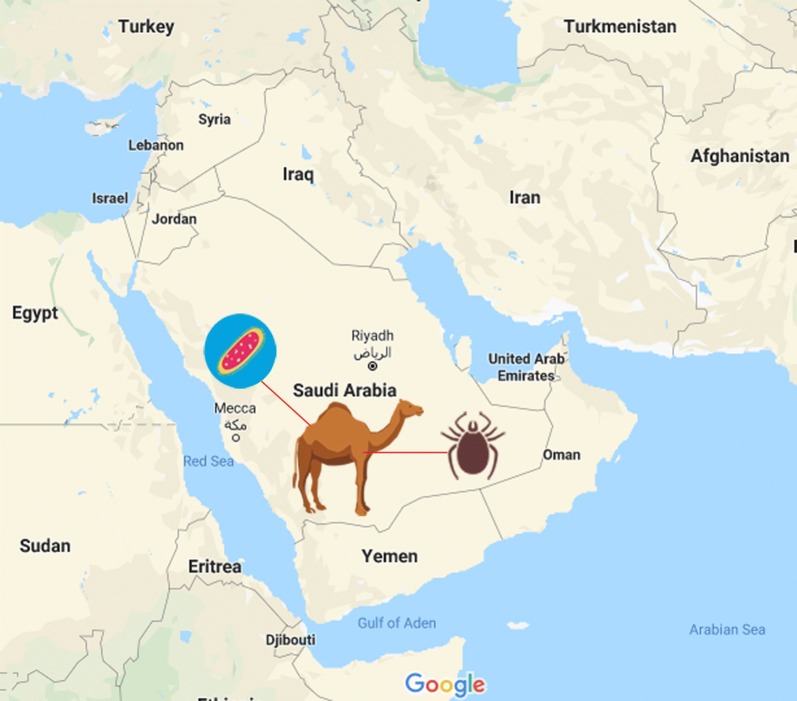

## Background

Ticks and transmitted tick-borne pathogens (TBPs) may cause a serious threat to humans, livestock, pets, and wildlife throughout the world [1, 2]. In addition to acting as the vectors of pathogens, ticks also affect the wellbeing of livestock directly through irritating bites, blood loss, damage to the skin and anorexia, leading to reduced growth [3]. Saudi Arabia is listed among the countries with a recent high growth in the camel population [4], having a population of approximately 500,000 in 2017 with the highest percentage in Riyadh Province [5]. The genus *Camelus* includes two species, *Camelus dromedarius* (Arabian camel or dromedary) distributed in North Africa and the Middle East, and *Camelus bactrianus* (Bactrian camel) in cold steppes and the deserts of Central Asia [6]. The dromedary camel plays an important role in the economy, especially in the culture of Arabian countries. Apart from being adapted to the harsh environment, these pseudo-ruminants, popularly known as “ship of the deserts” are multipurpose animals used for milk and meat production, hair/felt, racing, transportation and tourism [4, 6]. Camel production is severely affected by various diseases, especially in the absence of adequate veterinary services [7]. Many endo- and ectoparasites affect their health, productivity and performance including ticks [7], with more than 20 ixodid species found to infest camels [8, 9]. Among them, ticks of the genus *Hyalomma* are the most prevalent species [10, 11], which could act as vectors for *Theileria* spp. (i.e. *Theileria annulata* and *Theileria ovis*), *Babesia* spp. (i.e. *Babesia bigemina*, *Babesia caballi*, *Babesia ovis*) [12–15] and *Anaplasma* spp. [12]. Nonetheless, the role of *Hyalomma* spp. ticks as competent vectors of many of these pathogens is still uncertain.

Although genus *Anaplasma* includes six recognized species, *A. phagocytophilum* is the major zoonotic pathogen [16]. Apart from humans, *A. phagocytophilum* has been detected in dogs, horses, cats, sheep, goats, cattle and camels [17, 18]. In addition, three new possible *Anaplasma* species, *Anaplasma odocoilei* [19], *Anaplasma capra* [20] and “*Candidatus* Anaplasma camelii” [21] have recently been reported from deer, goats and camels, respectively. Being largely imported from neighboring countries, livestock may serve as a source of pathogens to camels in Saudi Arabia [22]. Conventional microscopic examination revealed the presence of TBPs such as *Anaplasma* spp., *Babesia* spp. and *Theileria* spp. in camels of Saudi Arabia [23–25]. However, knowledge of TBPs in camels of this country is very limited with few molecular epidemiological studies conducted on a limited number of animals [15, 26]. Therefore, the present study aimed to determine the prevalence of ixodid ticks and molecularly investigate their associated pathogens in camels from Saudi Arabia.

## Methods

### Sampling procedures

From March to September 2018, a total of 170 camels were screened to assess the intensity of tick infestation and the prevalence of TBPs. Camels came from Riyadh Province (24°0′N, 45°30′E), the central part of Saudi Arabia. Each camel was apparently healthy at the time of sampling and was screened for tick infestation. Ticks found within 15 min were collected (2–5 ticks/infested animal), placed in labeled tubes individualized per camel, containing 70% ethanol. Ticks were identified to the species level by using morphological keys and descriptions [27–34]. Categorical data on age and sex was also collected from each camel. Approximately 2 ml of blood was collected from the cephalic vein of camels and preserved in K_3_EDTA coated vacutainer tubes (BD Vacutainer^®^ Tube, BD Diagnostic Systems, Melbourne, Australia) until DNA extraction.

### DNA isolation from camel blood, molecular analysis by PCR and sequencing

Genomic DNA was isolated from whole blood samples using the Wizard® Genomic DNA Purification Kit (Promega, Madison, WI, USA), following the manufacturer’s instructions and was stored at − 80 °C. All DNA samples were tested for the presence of apicomplexan (i.e. *Babesia* spp., *Theileria* spp. and *Hepatozoon* spp.) and rickettsial parasites (i.e. *Ehrlichia* spp. and *Anaplasma* spp.) by conventional PCR (cPCR) using primers targeting partial 18S rRNA and 16S rRNA genes, as described previously [35–38] (Table 1). Initially, a single PCR reaction was used for the simultaneous detection of apicomplexan and rickettsial pathogens. Individual species-specific PCRs were then performed (Table 1) in the positive samples to assess the co-infections with more than one parasite species. For all reactions, DNA of pathogen-positive blood samples served as a positive control. Amplified PCR products were examined on 2% agarose gels stained with GelRed (VWR International PBI, Milan, Italy) and visualized on a GelLogic 100 gel documentation system (Kodak, New York, USA). The PCR products were purified and sequenced in both directions using the same forward and reverse primers, employing the Big Dye Terminator v.3.1 chemistry in a 3130 Genetic analyzer (Applied Biosystems, California, USA) in an automated sequencer (ABI-PRISM 377). Gene sequences were edited, aligned and analyzed using the Geneious platform version 9.0 (Biomatters Ltd., Auckland, New Zealand) and compared with the available sequences in GenBank using the Basic Local Alignment Search Tool (BLAST; http://blast.ncbi.nlm.nih.gov/Blast.cgi).

**Table 1:** Primers and target genes of pathogens investigated

Pathogens	Primers (5′-3′)	Target gene	Product size (bp)	Cycling conditions	Reference
*Babesia* spp./*Theileria* spp.	RLBF: GAGGTAGTGACAAGAAATAACAATA	18S rRNA	460	95 °C—600 s, 95 °C—30 s, 52 °C—30 s (× 40), 72 °C—60 s, 72 °C—420 s	[35]
	RLBR: TCTTCGATCCCCTAACTTTC				
*Babesia* spp.	PiroA: AATACCCAATCCTGACACAGGG	18S rRNA	410	95 °C—600 s, 95 °C—30 s, 62 °C—30 s (× 35), 72 °C—30 s, 72 °C—420 s	[36]
	PiroB: TTAAATACGAATGCCCCCAAC				
*Hepatozoon canis*	HepF: ATACATGAGCAAAATCTCAAC	18S rRNA	625	95 °C—600 s, 95 °C—30 s, 60 °C—30 s (× 35), 72 °C—60 s, 72 °C—300 s	[37]
	HepR: CTTATTATTCCATGCTGCAG				
*Ehrlichia* spp*./ Anaplasma* spp.	EHR16SD: GGTACCYACAGAAGAAGTCC	16S rRNA	345	95 °C—120 s, 94 °C—60 s, 54 °C—30 s (× 40), 72 °C—30 s, 72 °C—300 s	[38]
	EHR16SR: TAGCACTCATCGTTTACAGC				

### Phylogenetic analysis

Phylogenetic relationships were inferred using the Maximum Likelihood (ML) method based on the Kimura 2-parameter model [39], Hasegawa–Kishino–Yano model [40] with the Gamma distribution (+G) were used to model evolutionary rate differences among sites selected by the best-fit model [41]. Evolutionary analysis was conducted on 8000 bootstrap replications using the MEGA X software [42]. Homologous sequences from *Adelina bambarooniae* and *Wolbachia pipientis* were used as the outgroups (GenBank: AF494058 and AF179630, respectively).

### Statistical analysis

Prevalence (i.e. proportion of hosts infested by ticks), tick infestation burden (i.e. arithmetic mean count of ticks on each infested host) and pathogen infection rates were assessed using Quantitative Parasitology software (version 3.0) [43].

## Results

Of the 170 camels examined, 116 (68.2%; 95% CI: 60.9–75.1%) were infested by 296 ticks (mean intensity of 2.53; 95% CI: 2.4–2.6), with 206 (69.6%) being males and 90 (30.4%) females. All ticks were morphologically identified as belonging to the genus *Hyalomma*, with the most representative tick species being *H. dromedarii* (76.4%), followed by *Hyalomma impeltatum* (23.3%) and *Hyalomma excavatum* (0.3%).

Data on sex and age of sampled camels along with the number and percentage positivity for TBPs are reported in Table 2. Out of 170 camels tested, 13 (7.6%; 95% CI: 4.3–12.8%) were positive for at least one pathogen with *A. platys* being the highest prevalent pathogen (5.3%; 95% CI: 2.7–9.9%), followed by *A. phagocytophilum*, *Anaplasma* sp., *E. canis* and *H. canis* (0.6% each; 95% CI: 0.04–3.4%). None of the camels were found to be co-infected. All samples tested were negative for piroplasmids (*Babesia* spp. and *Theileria* spp.).

**Table 2:** Prevalence of infection among camels with tick-borne pathogens according to sex and age

Category	*Hepatozoon canis*	*Ehrlichia canis*	*Anaplasma platys*	*Anaplasma phagocytophilum*	*Anaplasma* sp.	Total
	Positive (%)	Positive (%)	Positive (%)	Positive (%)	Positive (%)	Positive (%)
Sex						
Male (*n* = 56)	–	1 (1.8)	3 (5.4)	–	–	4 (7.1)
Female (*n* = 114)	1 (0.9)	–	6 (5.3)	1 (0.6)	1 (0.6)	9 (7.9)
Age						
≤ 1 year (*n* = 18)	–	–	1 (5.6)	1 (5.6)	1 (5.6)	3 (16.7)
1–5 years (*n* = 106)	1 (0.9)	–	3 (2.8)	1 (0.9)	–	5 (4.7)
6–15 years (*n* = 46)	–	1 (2.2)	5 (10.9)	–	–	6 (13)

Representative sequences for each pathogen displayed 99.7–100% nucleotide identity with those available in the GenBank database. Two sequence types (ST) were identified for *A. platys* (ST1, *n* = 6, 100% identity with KX818218; ST2, *n* = 3, 99.7% identity with KX792011). One ST each for *A. phagocytophilum* (99.8% identity with MN648675), and *Anaplasma* sp. (99.7% identity with MN317255). One ST was identified for *H. canis* (100% identity with MK673842) and for *E. canis* (100% identity with KP182942), respectively.

**Fig. 1:**
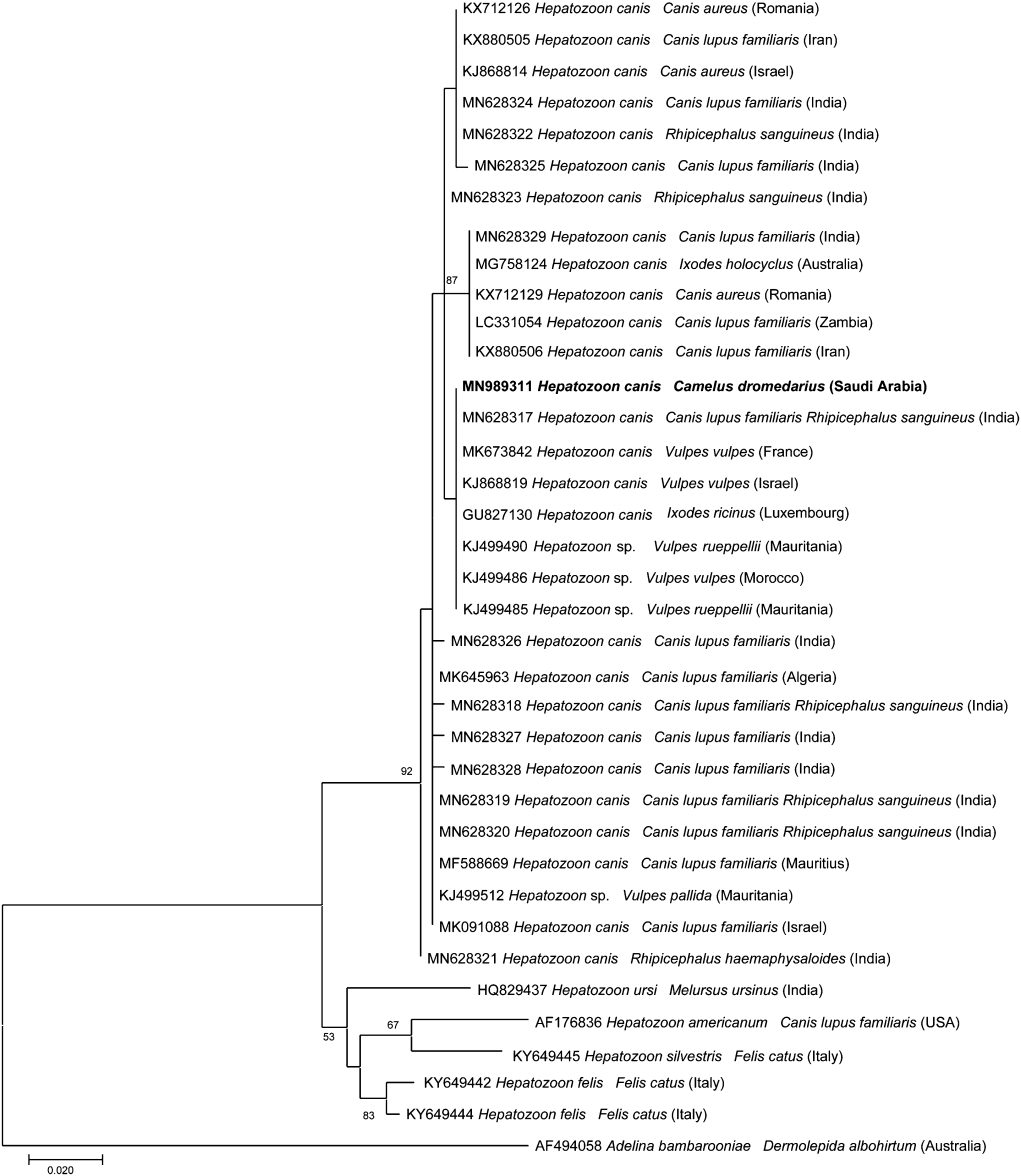
Phylogenetic relationships of *Hepatozoon canis* sequence detected in this study and other *Hepatozoon* spp. based on a partial sequence of the 18S rRNA gene. The analyses were performed using a maximum likelihood method with Hasegawa–Kishino–Yano model. *Adelina bambarooniae* (GenBank: AF494058) was used as the outgroup. Sequences are presented by GenBank accession number, host species and country of origin

**Fig. 2:**
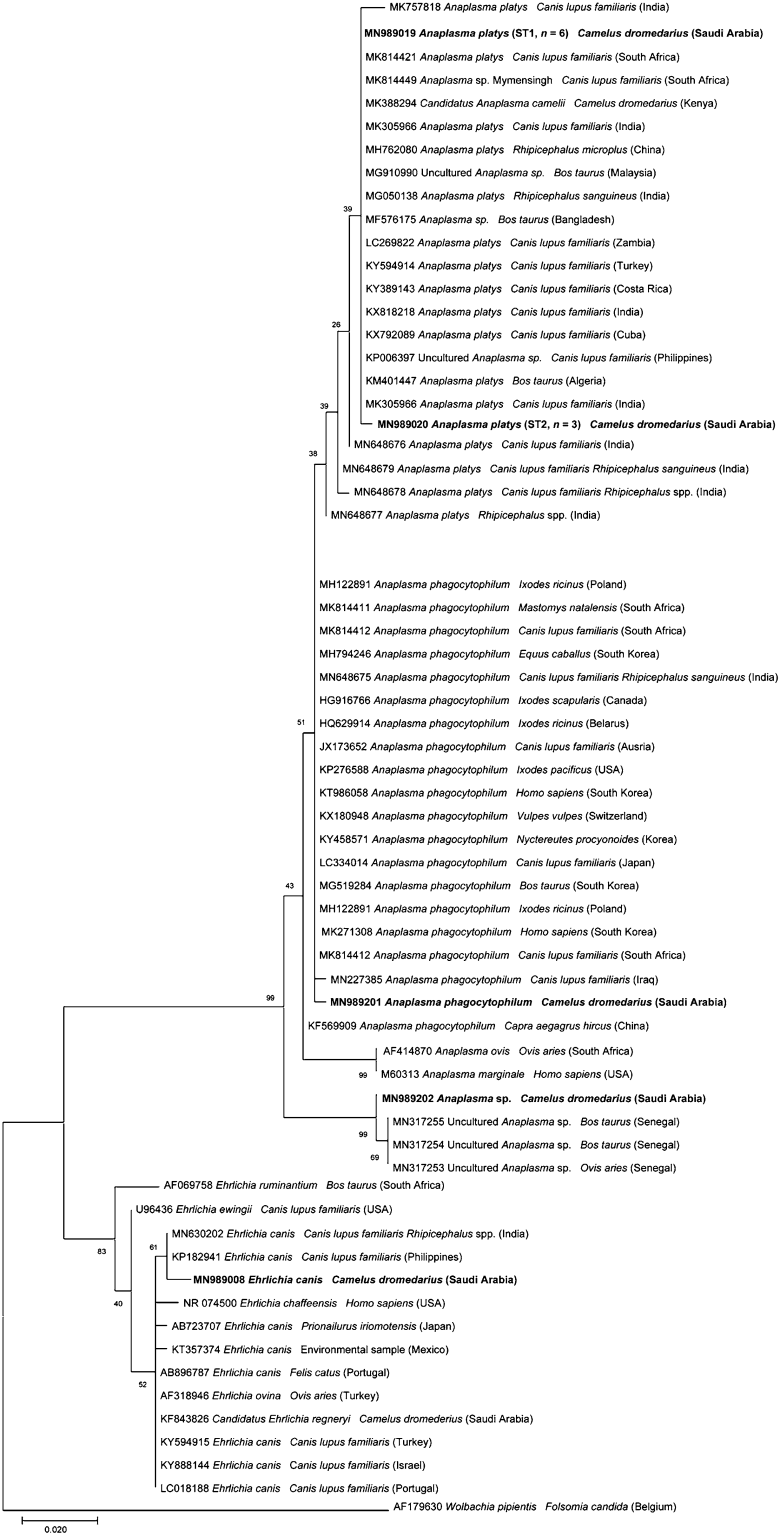
Phylogenetic relationships of *Anaplasma* spp. sequence types (*Anaplasma platys*, *Anaplasma phagocytophilum* and *Anaplasma* sp.) and an *Ehrlichia canis* sequence detected in this study and other *Anaplasma* spp. and *Ehrlichia* spp. based on a partial sequence of the 16S rRNA gene. The analyses were performed using a maximum likelihood method with Kimura 2-parameter model. *Wolbachia pipientis* (GenBank: AF179630) was used as the outgroup. Sequences are presented by GenBank accession number, host species and country of origin

Molecular identification of representative STs for *H. canis*, *E. canis* and *Anaplasma* spp. were supported by the distinct separation of species-specific clades, inferred from the phylogenetic analyses (Figs. 1, 2). In the ML tree, the representative ST of *H. canis* clustered within a well-supported clade including sequences of *H. canis* from wild canids and differing from other *Hepatozoon* spp. (Fig. 1). Rickettsiales herein detected (i.e. *A. platys*, *A. phagocytophilum*, *Anaplasma* sp., and *E. canis*) were included in two robust clades of the ML tree (Fig. 2). In particular, the ST of *E. canis* clustered in the clade including those of different hosts from different geographical regions (Fig. 2). Among *Anaplasma* spp., both STs of *A. platys* and of *A. phagocytophilum* were included in the corresponding species-specific paraphyletic clade (Fig. 2) whilst *Anaplasma* sp. clustered within the sister clade, which included sequences of *A. marginale* and *A. ovis* (Fig. 2).

Representative sequences of pathogens detected in this study were deposited in the GenBank database under the accession numbers MN989008 (*E. canis*), MN989019 and MN989020 (*A. platys*), MN989201 (*A. phagocytophilum*), MN989202 (*Anaplasma* sp.) and MN989311 (*H. canis*).

## Discussion

The high prevalence of tick infestation (68.2%) and the circulation of TBPs (7.6%) among camels in Saudi Arabia represents a risk to the health and welfare of these animals. Being blood-sucking arthropods, ticks can cause irritation and traumatic injuries to the skin of camels. The damaged skin will adversely affect the energy and water balance of camels in arid environment [44] and also attract flies leading possibly to myiasis infections [45]. The most prevalent tick species identified was *H. dromedarii*, which is considered as the main species parasitizing dromedary camels [10, 11]. *Hyalomma dromedarii* is a thermophilic tick usually found in arid and hyper-arid regions [46] with the high prevalence reported from camels in Sudan, Iran, Egypt, Saudi Arabia and Tunisia, with an infection rate ranging between 49–89% [10, 46–49] although it can also infest sheep, goats and horses [50]. This tick species is the principal vector of *Theileria* spp. of domestic and wild ungulates in Saudi Arabia [8]. The other two species herein identified in camels, *H. impeltatum* and *H. excavatum*, usually parasitize cattle and sheep [8, 51] and their finding in camels might be due to the husbandry practices in desert areas where all livestock share common inhabitancy, wandering in nature searching for water sources and grazing land.

The absence of *Babesia* spp. and *Theileria* spp. DNA in tested samples agrees with previous studies [13, 15] though these pathogens were diagnosed on some occasions by microscopic examination [23–25]. However, these results do not allow drawing any definitive conclusions about the occurrence of those pathogens in the sampled population, also considering the temporary nature of parasitemia in the blood of infected animals. To date, DNA of *Theileria equi*, *T. annulata*, *T. mutans*, *T. ovis* and *B. caballi* have been detected in blood of dromedaries [18, 52–55]. There is limited knowledge on piroplasms specific for camels and due to lack of experimental infections and molecular characterisation, the taxonomic status of some species such as *Theileria camelensis* [56], *Theileria dromedarii* [57], *Theileria assiutis* [58] and *Babesia cameli* [59] remain unresolved. The detection of *H. canis* in one camel represents, to our knowledge, the first report of this pathogen among camels, and this could be accounted for by the low host specificity and ubiquitous distribution of *H. canis* [60] and its vectors (i.e. *Rhipicephalus sanguineus* (*sensu lato*)). While *R. sanguineus* (*s.l*.) was not found on camels in this study, this tick is known to occur on dogs in Riyadh [61].

Among rickettsial organisms, *A. platys* was the most prevalent pathogen (*n* = 9, 5.3%), though a much higher prevalence of *Anaplasma* spp. was detected in previous studies (i.e. 26% from Saudi Arabia [21] and 61% from Nigeria [55]). *Anaplasma platys* is a parasite with tropism for platelets having a wide host range, primarily being the causative agent of canine cyclic thrombocytopenia [62]. Even though definitive proof of the vector competence of *R. sanguineus* (*s.l*.) is currently lacking, this tick species is supposed to be the vector of *A. platys* [63]. Indeed, the presence of *A. platys* DNA amplified from *R. sanguineus* (*s.l*.) collected from Bactrian camels has been previously reported [64]. Although *A. platys* was initially considered to be a pathogen of dogs, recent reports support the occurrence of this pathogen in other livestock and humans suggesting a more broader host range for this pathogen [55]. Accordingly, *E. canis* mainly found in dogs, has been reported in domestic ruminants [65], with some strains diagnosed in dromedary camel of Saudi Arabia [21]. The occurrence of canine pathogens such as *A. platys* and *E. canis* in camels can be due to the co-inhabitance of these animals in desert area as well as to the strict affiliation of *R. sanguineus* (*s.l*.) to canids and its ability in surviving a large array of environmental conditions [66]. Overall these ecological features give a hint about the possibility of transmission of these pathogens from dogs to camels.

For its zoonotic potential, the retrieval of *A. phagocytophilum* in camelids is relevant. This pathogen has been mostly diagnosed worldwide in wild roe deer and a wide variety of wildlife fauna [67–69]. In camels, relatively high *A. phagocytophilum* positivity values have been reported in Tunisia (i.e. 29.2% based on serology) [70] and Iran (34.3% based on PCR) [71]. While it has been demonstrated that several animal species may act as reservoirs of *A. phagocytophilum* [72, 73], the role of camelids remains to be ascertained. In the same way, the competence of *Hyalomma* spp. ticks as vectors for this pathogen needs confirmation.

Sequence analysis of the data revealed the circulation of two different STs of *A. platys* while pathogens like *H. canis* and *E. canis* had only one ST. High genetic variability has been already reported within *Anaplasma* spp. in different hosts from different geographical locations [21, 74]. In the ML tree, two STs of *A. platys* from camels clustered within those of dogs irrespective of the geographical location, indicating its circulation amongst different animal species. This may occur due to a spillover of *A. platys* infection from canids to camelids [55]. Moreover, a ST of *Anaplasma* sp. found herein clustered with a group of *Anaplasma* spp. sequences from other ruminants from Senegal. This strengthens the possibility of genetic variation and high diversity of *Anaplasma* spp. The phylogenetic analysis showed that *H. canis* from camel clustered with those of wild carnivores (i.e. red foxes and of Ruppell’s foxes) in a separate sister clade. Nonetheless, the finding of this parasite in a camel is probably a casual finding in an accidental host.

## Conclusions

Our data indicate that *H. dromedarii* is the most prevalent tick infesting camels from Saudi Arabia and that these animals are exposed to many TBPs. The identification of pathogens such as *A. platys*, *A. phagocytophilum*, *E. canis* and *H. canis* not vectored by *Hyalomma* ticks suggests that further investigations should be carried out. It is advisable to undertake either molecular screening of the tick salivary glands or to perform transmission experiments using tick colonies, to obtain more reliable information on the vectoral role of these ticks. Since some of the detected pathogens are of zoonotic concern, adequate measures must be taken for the regular surveillance and control of zoonotic pathogens in camels.

## Data Availability

All data generated or analyzed during this study are included in this published article.

## Abbreviations

CI: confidence interval
ML: maximum likelihood
ST: sequence type
*s*.*l*.: *sensu lato*
TBP: tick-borne pathogen
